# The epidemiology of pertussis in Germany: past and present

**DOI:** 10.1186/1471-2334-9-22

**Published:** 2009-02-25

**Authors:** Wiebke Hellenbrand, Dietmar Beier, Evelin Jensen, Martina Littmann, Christiane Meyer, Hanna Oppermann, Carl-Heinz Wirsing von König, Sabine Reiter

**Affiliations:** 1 Immunisation Division, Department of Infectious Disease Epidemiology, Robert Koch Institute, Seestrasse 10, 13353 Berlin, Germany; 2 Department of Hygiene and Environmental Medicine, Infectious Disease Epidemiology, Disease Reporting and Medical Microbiology, Landesuntersuchungsanstalt, Chemnitz, Zschopauerstr. 87, 09111 Chemnitz, Germany; 3 Thüringer Landesamt für Lebensmittelsicherheit und Verbraucherschutz (TLLV), Abt. Medizinaluntersuchung, Tennstedter Straße 8/9, 99947 Bad Langensalza, Germany; 4 Landesamt für Gesundheit und Soziales Mecklenburg-Vorpommern, Abteilung Gesundheit, Dezernat Infektionsschutz/Prävention, Gertrudenstr. 11, 18057 Rostock, Germany; 5 Agency for Consumer Protection of the Federal State of Saxony-Anhalt, Health Care Department, Wallonerberg 2-3, 39104 Magdeburg, Germany; 6 Institute for Hygiene and Laboratory Medicine, HELIOS Klinikum Krefeld, Lutherplatz 40, 47805 Krefeld, Germany

## Abstract

**Background:**

Current and past pertussis epidemiology in the two parts of Germany is compared in the context of different histories of vaccination recommendations and coverage to better understand patterns of disease transmission.

**Methods:**

Available regional pertussis surveillance and vaccination coverage data, supplemented by a literature search for published surveys as well as official national hospital and mortality statistics, were analyzed in the context of respective vaccination recommendations from 1964 onwards.

**Results:**

Routine childhood pertussis vaccination was recommended in the German Democratic Republic (GDR) from 1964 and in former West German states (FWG) from 1969, but withdrawn from 1974–1991 in FWG. Pertussis incidence declined to <1 case/100.000 inhabitants in GDR prior to reunification in 1991, while in FWG, where pertussis was not notifiable after 1961, incidence was estimated at 160–180 cases/100.000 inhabitants in the 1970s-1980s. Despite recommendations for universal childhood immunization in 1991, vaccination coverage decreased in former East German States (FEG) and increased only slowly in FWG. After introduction of acellular pertussis vaccines in 1995, vaccination coverage increased markedly among younger children, but remains low in adolescents, especially in FWG, despite introduction of a booster vaccination for 9–17 year olds in 2000. Reported pertussis incidence increased in FEG to 39.3 cases/100.000 inhabitants in 2007, with the proportion of adults increasing from 20% in 1995 to 68% in 2007. From 2004–2007, incidence was highest among 5–14 year-old children, with a high proportion fully vaccinated according to official recommendations, which did not include a preschool booster until 2006. Hospital discharge statistics revealed a ~2-fold higher pertussis morbidity among infants in FWG than FEG.

**Conclusion:**

The shift in pertussis morbidity to older age groups observed in FEG is similar to reports from other countries with longstanding vaccination programs and suggests that additional booster vaccination may be necessary beyond adolescence. The high proportion of fully vaccinated cases in older children in FEG suggests waning immunity 5–10 years after primary immunisation in infancy. The higher incidence of pertussis hospitalisations in infants suggests a stronger force of infection in FWG than FEG. Nationwide pertussis reporting is required for better evaluation of transmission patterns and vaccination policy in both parts of Germany.

## Background

An increase in the incidence of pertussis, particularly among older children, adolescents and adults, was observed since the 1980s in the United States and since the 1990s in Canada and several European countries, all with high childhood pertussis vaccination coverage [1-5]. Pertussis is most severe among infants, who have the highest risk of complications, hospitalization and death [6-8]. Although the disease is frequently milder among older children, adolescents and adults, a protracted course is the rule and complications such as pneumonia, urinary incontinence, weight loss and rib fractures may occur [9-12]. Several factors contribute to the persistent circulation of the infectious agent, *Bordetella pertussis*, despite immunization programs, the most important of which is waning of pertussis-specific immunity after about 7 to 20 years after natural infection and 4 to 12 years after immunization [13,14]. Furthermore, *B. pertussis *is highly contagious, with a basic reproductive number of 12–17 [15]. Because of the non-specific initial presentation of the illness and because the infection may not cause the full-blown clinical picture in immunized individuals or those with previous natural infection, the diagnosis may be delayed or overlooked, thereby leading to prolonged transmission. Thus the susceptibility to and transmission of the disease is influenced by current and past vaccination coverage, vaccination schedule, type of vaccine in use and social mixing patterns.

Vaccination recommendations, resulting vaccination coverage and the incidence of pertussis differed markedly in the former German Democratic Republic (GDR) and the former West Germany (FWG), with differences persisting for some time after reunification. Therefore, the aim of our paper is to compare the epidemiology of pertussis in the two parts of Germany to better understand patterns of disease transmission and optimize prevention efforts.

## Methods

Official pertussis vaccination recommendations in the GDR, FWG and the reunited Germany were reviewed. The Standing Committee on Vaccination (STIKO) was responsible for vaccination recommendations in FWG since 1972, and since 1991 also in the reunited Germany. Prior to 1972, a "Pertussis-Committee" of pediatric and infectious disease specialists made recommendations in FWG [16]. Recommendations are generally adopted by the individual federal states, which can, however, deviate from them. In the former GDR the ministry of health was responsible for making vaccination recommendations [16].

Data on pertussis vaccination coverage in Germany routinely obtained from obligatory medical examinations performed by the regional health authorities at school entry (children aged 5 to 7 years) have been made available to RKI by the federal states since 1998. In addition, Medline, Embase and Embase Alert data bases were searched for vaccination coverage studies in Germany using the free text search terms [(pertussis or whooping cough) AND Germany AND (vaccination coverage or vaccination rate)].

The following official data sources were used to obtain data on the incidence of pertussis: Pertussis was statutorily notifiable from 1947 to 1961 in Germany and data were published by the Federal Statistical Office [17]. In FWG, notification was no longer required as of 1962; thus surveillance data are lacking. In GDR pertussis was a statutorily notifiable disease and aggregate data for 1962–1990 were published by the Statistical Office of the German Democratic Republic. Reporting continued after reunification in five former East German states (referred to as FEG: Brandenburg (BB), Mecklenburg-Western Pomerania (MV), Saxony (SN), Saxony-Anhalt (ST), Thuringia (TH), but not East Berlin, counted as part of FWG after reunification in 1991). These 5 states transmitted case-based data with a minimal variable set to the Robert Koch-Institute (RKI), the federal institution responsible for disease control and prevention, from 1995 to 2000, enabling a basic demographic analysis. Pertussis was not made a notifiable disease under the new German Protection Against Infection Law implemented in 2001. However, FEG continued to require statutory reporting of pertussis according to state-specific regulations. From 2001 onwards notified case-based data were transmitted to RKI, although these were incomplete in 2001.

A uniform case definition was applied from 2002 onwards [18], thus a detailed analysis of FEG surveillance data was undertaken for the period January 2002 to December 2007 according to region, age and sex, hospitalization and vaccination status, as well as outbreaks. According to the surveillance case definition in place from 2002–2008, a clinical case must manifest at least one of the following symptoms for at least 2 weeks: i. cough attacks, ii. whooping, iii. post-tussive vomiting or – in infants – iv. episodes of apnea. Laboratory diagnosis, initiated at the discretion of the physician, consists of cultural isolation or detection of *B. pertussis *by PCR in nasopharyngeal swabs or secretions or serological diagnosis by means of an elevated pertussis-specific IgA- antibody concentration in a single serum sample or a 4-fold increase in IgG- or IgA- pertussis-specific antibodies using any commercially available test kit (e.g. Serion ELISA *classic *measuring IgG-[PT, FHA (filamentous hemagglutinin)] and IgA- (PT, FHA) antibodies, Genzyme Virotech GmbH ELISA measuring IgG- and IgA-antibodies against PT, or Bordetella pertussis IgG Virastripe^®^, an immunoassay also measuring IgG-antibodies against PT and FHA (produced by Viramed) [19]. The majority of the 18,080 cases notified to RKI between January 2002 and December 2007 were laboratory confirmed (93.8%); 1.9% fulfilled the clinical case definition and were epidemiologically linked to a laboratory confirmed case and 4.4% solely fulfilled the clinical case definition. Of the laboratory confirmed cases, 172 (1.0%) were diagnosed by culture, 1769 (9.8%) by PCR, 4861 (26.9%) by an increase in pertussis-specific antibodies, and 10,153 (56.2%) by a high concentration of pertussis-specific IgA-antibodies in a single serum sample. Detailed data on the vaccination status of cases were available from 2004 onwards for 78% of notified cases. A case was defined as adequately vaccinated if 3 doses of pertussis vaccine had been given with the third dose less than one year prior to disease onset or if at least 4 doses had been given with the last dose = 10 years prior to disease onset. Doses received within 3 weeks prior to disease onset were not counted.

Detailed hospital discharge and mortality data for pertussis (principal diagnosis and underlying cause of death, respectively) were provided by the German Federal Statistical Office http://www.gbe-bund.de/gbe10/isgbe.prc_impressum?p_uid=gast&p_aid=23980486&p_sprache=E.

In addition to official data on pertussis disease burden, Medline, Embase and Embase Alert data bases were searched for further sources on pertussis incidence, such as sentinel studies or regional surveys, in Germany using the search terms [(pertussis or whooping cough) AND Germany AND (incidence or disease burden or morbidity)].

## Results

### Vaccination recommendations prior to 1991

In GDR, pertussis vaccination was mandatory for all children from 1964 onwards [16]. In the 1980s three doses of whole cell vaccine were recommended at the age of 2, 3, and 6 months. A fourth dose was recommended in the third year of life [20]. Between 1971 and 1979 a fifth dose was recommended prior to school entry [21,22].

In FWG routine pertussis vaccination with whole cell vaccine was recommended for infants and toddlers from 1969 until 1974 [16]. As in some other countries, such as the UK, this recommendation was withdrawn by STIKO from 1974 [23] to 1991 due to anecdotal reports of severe adverse effects affecting the central nervous system. During this period, pertussis vaccination was only recommended for high risk children prior to their second birthday.

### Vaccination coverage prior to 1991

Vaccination coverage in preschool children (≥ 4 doses) was >90% in GDR during the 1980s [20]. Surveys and estimates based on vaccine sales data in the 1970s and 1980s in FWG revealed a childhood vaccination coverage of 50–60% in southern and 2–20% in northern regions [24].

### Pertussis incidence prior to 1991

**Figure 1:**
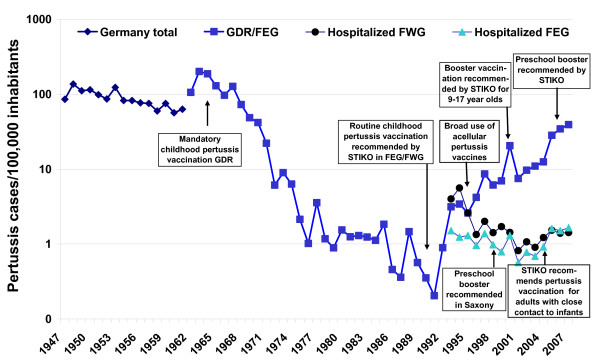
**Incidence of notified pertussis illness (1947–2007) and hospitalizations (1993–2007) in Germany, (GDR: German Democratic Republic, FEG: Former East Germany, FWG: Former West Germany)**.

Pertussis incidence based on reported cases in GDR declined to <1/100,000 population in the late 1980s prior to reunification [25,26] (Fig. 1). Incidence estimates in FWG based on regional surveys in the 1970s and 1980s ranged from 160–180 cases/100,000 population [25,27]. Among children <6 years, pertussis incidence was estimated at 4%–6% annually between 1987 and 1990 [28].

### Vaccination recommendations after 1990

In 1991 pertussis vaccination for all infants with whole-cell pertussis vaccine was again recommended by STIKO in the reunified Germany, with four vaccine doses at the ages of 2, 3, 4 and 11 to 14 months [29]. In 1993 STIKO recommended catch-up vaccination for all children up to their sixth birthday [30]. In 1995 acellular vaccines with 2 (pertussis toxin (PT) and filamentous hemagglutinin (FHA)), 3 (PT, FHA and pertactin (PRN)) or 5 components (PT, FHA, PRN and fimbriae 2 and 3) were licensed in Germany. These were exclusively recommended from 1997 onwards. In 2000 a single booster vaccination was recommended for children aged 9 to 17 years [31]. In 2003 STIKO recommended pertussis vaccination for child care workers and health care workers [32] and in 2004 additionally for adults with close contact to infants (parents and other care givers) prior to the birth of the infant (cocoon strategy) [33]. An additional booster dose at the age of 5–6 years was recommended by STIKO in January, 2006. In Saxony, the state committee on vaccination (SIKO) recommended a preschool booster already in 1998 and booster vaccinations for adults at 10 year intervals in 2007 [34].

### Vaccination coverage after 1990

Pertussis vaccine uptake increased only very gradually in FWG after 1991 prior to the broad introduction of acellular vaccines in 1995, which were exclusively recommended from 1998 onwards. In FEG vaccine uptake actually decreased between 1991 and 1995. An interview survey performed by Kirschner et al. in 1994–1995 [35] revealed a vaccination coverage at 2 years of age (at least 4 vaccine doses) of 8.8% (children born in 1989) and 46.6% (children born in 1992) in FWG and 65.5% (children born in 1989) and 46.3% (children born in 1992) in FEG. Of children born in 1992, 92.5% had begun the vaccination series in FEG but only 76.7% in FWG. From 1998 until 2007, vaccination coverage of children at school entry increased steadily in FEG from 85.8% to 95.9% and in FWG from 57.7% to 92.2%.

Vaccination coverage was also assessed in the recent German Health Interview and Examination Survey for Children and Adolescents (KiGGS), which examined a representative sample of 17,461 children from 0 to 17 years between May 2003 and May 2006, of whom 16,460 (93.1%) presented their vaccination record [36,37]. Pertussis vaccination coverage (at least 4 vaccine doses) was 84.9% (95% CI: 81.7%–87.6%) at the age of 2 years and peaked at 90.4% (95% CI: 89.2–91.5%) in 3 to 6 year old children (FEG: 91.9% (95% CI: 89.9–93.5%), FWG: 90.1 (95% CI: 88.7–91.3%). In older children, vaccination coverage decreased markedly with age to 36.1% (95% CI: 32.0–40.4%) in 14 to 17-year old adolescents, who had a significantly higher vaccination coverage in FEG (FEG: 78.8% (95% CI: 73.3%–83.5%) than FWG: 23.1% (95%CI: 20.0%–26.4%). Adolescents aged 14–17 years had also received the recommended booster vaccination more frequently in FEG (39.5% (95% CI: 34.6%–44.7%)) than in FWG (13.3% (95% CI: 11.0%–16.0%)). Only 10% of 14–17 year olds not vaccinated at a younger age had received at least one dose of pertussis-containing vaccine. In Saxony, vaccination coverage with a 5^th ^vaccine dose in 7–8 year-old children reached 71.5% in 2006 (unpublished data DB). Vaccination coverage data for the preschool booster recommended in 2006 in all other states are unavailable thus far.

### Pertussis incidence in former East German States after 1990 based on surveillance data

**Figure 2:**
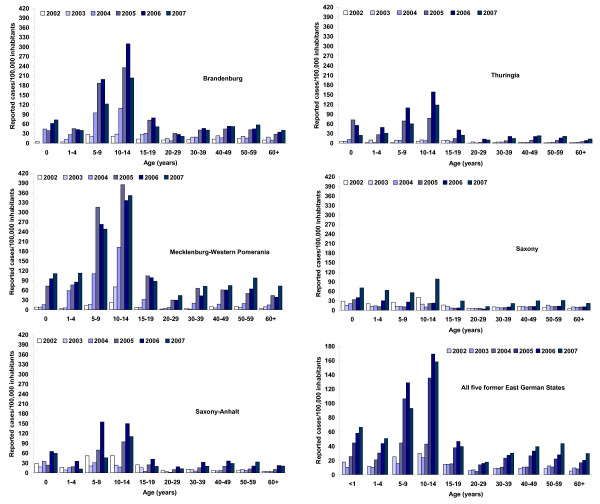
**Pertussis incidence in former East German States 2002–2007 based on notification data according to state-specific infectious disease surveillance laws**.

Decreasing vaccination coverage after reunification in the early 1990s in FEG was associated with an increase in pertussis incidence to 3.4 cases/10000 inhabitants in 1994 (Fig. 1). Following a slight decrease in incidence from 1995 to 1997, pertussis incidence increased further, despite the STIKO recommendation for a booster vaccination at the age of 9 to 17 years in 2000 [38]. In 2007 pertussis incidence in FEG climbed to 39.3 notified cases/100.000 inhabitants. Regionally, the increase in incidence was less marked and occurred later in Saxony-Anhalt (ST) and TH as compared to BB and MV (Fig. 2). The increase in incidence after 2002 was most evident in children aged 5 to 9 and 10 to14 years, among whom incidence was highest, reaching >300 cases/100,000 inhabitants in BB and MV (Fig. 2). However, the incidence increased in infants (>3-fold in the five states) and adults as well. In SN, the overall incidence was lowest and a marked increase in incidence – most pronounced among 10–14 year olds – did not occur until 2007 (Fig. 2). The proportion of notified pertussis cases reported as hospitalized from 2002–2007 was lowest in BB (1.9%) and MV (2.2%) and higher in ST (4.9%), TH (4.1%) and SN (4.6%). Hospitalization was reported for 39.5% of infants <1 year, 4.4% for 1–4 year old children, 1.9% of 5–59 year old individuals and 5.8% of persons ≥ 60 years.

### Sex and age distribution

Available surveillance data from 1995 onwards in FEG consistently showed a higher pertussis incidence in females (overall 60% of cases) than in males. This difference was mainly due to a higher proportion of females among adult cases.

**Figure 3:**
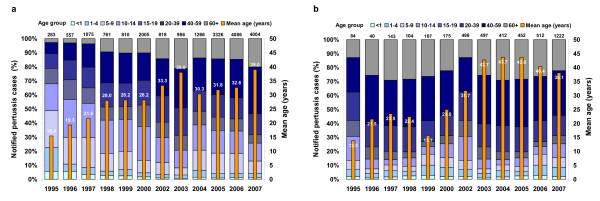
**Age distribution of notified pertussis cases in the former East German States Brandenburg, Mecklenburg Western Pomerania, Saxony Anhalt and Thuringia (Fig. 3a), and Saxony (Fig. 3b) 1995–2007**. The total number of cases is shown on top of the bars. Age-specific data for 2000 are incomplete and no age specific data are available for 2001.

An analysis of the age distribution of cases from 1995 to 2007 in the FEG states reveals a marked decrease in the proportion of cases among under 5-year olds, and, until 2003, also a decrease in the proportion of cases among older children and adolescents. While this development was largely sustained in Saxony until 2006, in the other 4 FEG states the proportion of cases among school children (5 to 14 year olds) increased markedly in 2004 (Fig. 3a &3b). The age distribution also shows that the absolute burden of disease is currently greatest in adults. In Saxony, the proportion of adults (>19 years of age) among all cases increased from 16.7% in 1995 to 80.3% in 2005 (subsequently decreasing 67.8% in 2007), while in the other 4 FEG States this proportion increased more gradually from 21.1% in 1995 to 68.7% in 2007, also reflected by the increase in mean age of reported pertussis cases over time (Fig. 3a &3b)

### Vaccination status of pertussis cases reported in former East German States, 2004–2007

**Figure 4:**
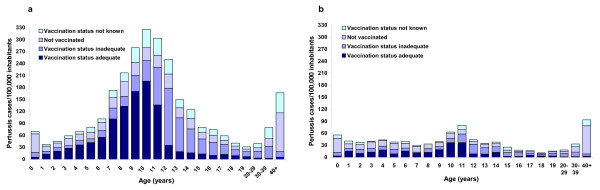
**Incidence of notified pertussis cases in the former East German States Brandenburg, Mecklenburg Western Pomerania, Saxony Anhalt and Thuringia (Fig. 4a), and Saxony (Fig. 4b), 2004–2007, according to age and vaccination status**.

FEG surveillance data from 2004 onwards reveal a high proportion of adequately vaccinated cases among children aged 6 to 11 years (Fig. 4). Among notified cases with information on vaccination status in BB, MV; ST and TH, the proportion of cases aged 6 to 11 years with ≥4 previous vaccine doses was 59%, 61%, 67% and 68% in 2004 to 2007 respectively, while this was 13%, 22%, 30% and 63% in SN (80/99 adequately vaccinated cases from Saxony were reported in 2007). In BB, MV, ST and TH, only 5.3% (73/1384) of these cases had been vaccinated within the 3 years preceding their illness; while in SN, this applied to 27.3% (27/99) of vaccinated cases, most of which were diagnosed in 2007 (20/27, all by PCR). Of all 4842 cases with at least one documented dose of pertussis vaccine in FEG from 2004–2007, 746 (15.4%) had been vaccinated < 3 years prior to illness onset (4.1% of all notified cases).

### Outbreaks

From 2002 to 2007, 16.0% of notified pertussis cases in FEG were reported as epidemiologically linked; 9.4% occurred in clusters of 5 or more cases. The largest cluster that occurred in MV in 2005–2006 consisted of 80 notified cases, but active case-finding during an outbreak investigation identified a further 24 persons who fulfilled the clinical case definition [19]

### Pertussis morbidity in both parts of Germany based on hospital discharge statistics, mortality data and sentinel surveillance in adults

**Figure 5:**
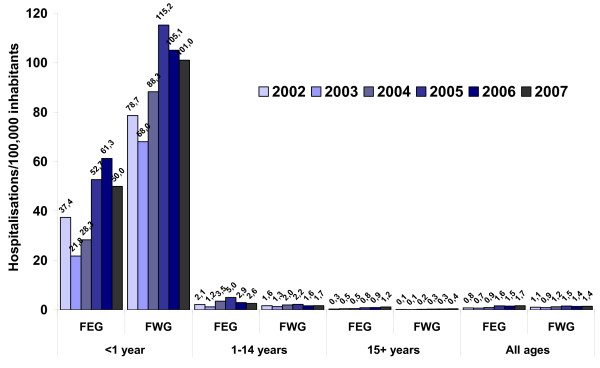
**Hospitalizations for pertussis per 100.000 inhabitants according to age and residence in former East (FEG) and West (FWG) Germany, 2002–2007 (Source of data: Statistics of hospital diagnoses, Federal Statistical Office, available at http://www.gbe-bund.de/gbe10/abrechnung.prc_abr_test_logon?p_uid=gast&p_aid=&p_sprache=D&p_knoten=VR&p_suchstring=pertussis**.

Hospital discharge statistics show a decrease in pertussis hospitalizations in FEG from 1993 to 2001 with some fluctuation, decreasing from 1.5 pertussis related hospitalisations/100.000 population in 1993 to 0.6 in 2001, only to increase again to 1.7 in 2007 (Fig. 1). The number of hospitalizations in FWG decreased from 5.6/100000 population in 1993 to a low of 0.8 in 2001, increasing again to 1.5 in 2005 (Fig. 1). Between 2002 and 2007 the proportion of all pertussis-based hospitalizations among infants decreased from 66.6% in 2002 to 59.7% in 2007 in FWG, while it fluctuated between 22.5% (2007) and 28.9% (2006) in FEG, with a much higher incidence of infant hospitalizations in FWG (Fig. 5). Among older children and adults, however, incidence of hospitalization for pertussis was slightly higher in FEG than FWG. From 2002–2007 in FEG, 69.1% more cases were recorded in the German Hospital Discharge Statistics (n = 959) than cases notified as having been hospitalized (n = 567) in the statutory surveillance system. Until 2007, the incidence of hospitalized pertussis cases aged <1 year based on hospital discharge statistics was higher than the total incidence based on notified cases in FEG (see Fig. 2 and Fig. 5).

Between 1970 and 2007 nine deaths attributed to pertussis were reported in FEG, four of which occurred since 2002, all in elderly adults [39]. In FWG, 231 deaths were reported in this period, with mortality gradually decreasing. The last three deaths were reported in 2001 in an infant, in 2005 in an elderly woman and the third in 2007 in a teenager [39]. Mortality is depicted in Figure 1.

Sentinel surveillance in general physician and internist practices for cough illness lasting at least 7 days in adults (≥ 18 years) was performed in the FEG city of Rostock and the FWG city of Krefeld from 2001 to 2004 [40]. Among the 971 patients recruited to the study, 10% had pertussis based on a positive PCR of nasopharyngeal aspirate and/or positive serology (elevated IgA- and IgG-antibodies against pertussis toxin and filamentous haemagglutinin). The incidence of pertussis based on this study was estimated at 169 cases/100,000 inhabitants per year in Krefeld and 160/100.000 inhabitants per year in Rostock, with an estimated 110.000 cases occurring in adults annually in Germany. This was 19-fold higher than the mean incidence in adults based on routine surveillance in FEG from 2002–2004 of 8.5 cases/100,000 inhabitants respectively.

See additional file 1 for summary of the development of surveillance practices, vaccination recommendations, vaccination coverage and disease burden in the two parts of Germany.

## Discussion

This overview describes markedly different development of pertussis epidemiology in the two parts of Germany prior to reunification in 1991. The divergent vaccination scenarios in the 1970s and 1980s led to a much lower pertussis incidence in FEG than FWG. These differences can be ascribed to limited use of pertussis vaccine in FWG due to concerns about adverse effects in contrast to continued high vaccination coverage in FEG. Despite the fact that severe adverse effects due to pertussis whole cell vaccine were never substantiated, reports to this effect led to reduced vaccine uptake and an increase in pertussis morbidity in other countries as well [41].

Analysis of pertussis epidemiology in Germany is limited in that notification data after 1961 are accessible only from FEG. Only a partial assessment of the situation in FWG is possible based on hospital discharge and mortality data. Moreover, the higher incidence of pertussis cases in FEG in infants based on hospital discharge statistics than based on notified cases until 2007 suggests a significant degree of underreporting in the FEG statutory surveillance system. Results of sentinel surveillance in a FEG city [40] also suggest that disease burden in adults is markedly underestimated by routine surveillance data, likely due to atypical presentation in persons with pre-existing natural or vaccine-induced immunity. A further potential limitation of routine surveillance data from FEG lies in the high proportion of cases diagnosed solely serologically. A recent study showed that the performance of the most frequently used ELISAs in Germany was variable regarding antigens used for testing, sensitivity (60–95%), and quantitative measurement of antibody levels [42]. Specificity was not reported, but other studies have shown high specificities of >90% for ELISAs based on PT- and FHA-antigens [43,44]. The observation that only 4.1% of all notified cases received a dose of pertussis vaccine < 3 years prior to illness onset suggests that recent vaccination is not an explanation for positive serology in the majority of cases. As well, an analysis of only those cases diagnosed by culture, PCR or a rise in antibody concentrations showed similar age distributions and trends over time as did the analysis of all cases presented here (data not shown).

In addition, the observed epidemiologic patterns, such as the higher pertussis incidence observed in women than men in FEG, are consistent with other studies: High pertussis-specific IgG-antibodies indicative of recent infection were found more frequently among women than men in a seroepidemiological study in England and Wales [45], and in a household contact study performed in Germany, secondary pertussis cases also occurred more frequently among women than men [46]. Furthermore, the recent increase in pertussis incidence in FEG associated with a shift to older age groups mirrors similar developments in pertussis epidemiology in other western countries with longstanding vaccination programs [47]. For instance, in the United States, an increase in incidence was observed beginning in the 1980s and, starting in the early 1990s, particularly among 10 to 19 year olds [10,48,49] despite improved vaccination uptake of the preschool booster [49], thus suggesting waning immunity 5–10 years after the preschool booster.

Correspondingly, despite high vaccination coverage with the primary vaccination series among FEG preschool children in recent years, the incidence of pertussis continued to increase in the four FEG states that did not recommend a booster vaccine dose prior to school entry until 2006. The increase in incidence since 2004 was most marked in children between 5 and 14 years of age, a high proportion of whom had received 4 doses of pertussis vaccine in infancy, suggesting that waning immunity was playing a role. This is supported by an outbreak investigation performed in a highly vaccinated school population in Mecklenburg Western-Pomerania in 2006, which showed a markedly higher attack rate in children who had received their fourth vaccine dose >5 years previously [19]. Increased vaccination coverage may lead to a period of decreased natural boosting, which may "unmask" the effect of waning vaccine-induced immunity, causing incidence to increase despite a lower level of transmission. Modeling studies suggest that the severity of disease – and thus the diagnostic threshold – depends on the level of transmission, with a higher incidence of severe disease expected at intermediate transmission intensities [50]. Thus an overall reduction in transmission initiated through increased vaccination coverage could theoretically lead to a higher incidence of more severe – and therefore recognized – disease through a combination of waning immunity and less natural boosting.

An increase in pertussis incidence in Saxony, where a preschool booster was recommended since 1998, occurred in 2007. Although all age groups were affected, the increase was most pronounced in children aged 10 to 14 years, suggesting that uptake of the adolescent booster vaccination may be insufficient or occurring too late. The Saxony state health authority also increased awareness for the diagnosis of pertussis among physicians by actively informing about the possibility of PCR diagnosis at the state-run microbiological laboratory in 2007 (personal communication, DB). This, along with the higher proportion of cases diagnosed < 3 years after the primary vaccination series in 2007, suggests that a change in diagnostic practices might partially explain the observed increase. The incidence in Saxony still remains lower than that in all other FEG states (Fig. 2) and the increase in 2007 may also in part be due to periodic variation in incidence.

The continued increase in pertussis incidence in FEG despite improved vaccine uptake after the mid-1990s also coincided with widespread introduction of acellular vaccines in Germany. Thus another possible explanation might be a shorter duration of immunity compared to vaccination with whole cell vaccines. In FEG, the pertussis component of the combined diphtheria, tetanus, pertussis vaccine consisted of alum-adsorbed *B. pertussis *(30–40 × 10^9 ^bacteria/ml), equivalent to at least 4 international potency units per 0.5 ml dose [20,51], fulfilled international potency requirements of at least 4 IPU issued by the World Health Organization in 1964 [52]. In general, available studies suggest a similar duration of immunity after vaccination with acellular and whole cell vaccines [53], although comparison is difficult due to the heterogeneity of both vaccine types, and inability to control for circulating levels of pertussis in the population, which could influence immunity through natural boosting.

The higher incidence of hospitalization due to pertussis in infants in FWG than FEG suggests a higher level of disease transmission in FWG, in keeping with lower vaccination coverage particularly in older children and adolescents, who along with adults presumably act as a transmission reservoir for infants [54-58]. However, the slightly higher incidence of pertussis-related hospitalizations in FEG in older children and adolescents, who have higher vaccination coverage than their counterparts in FWG, is surprising. A higher incidence of pertussis hospitalizations in FEG than FWG was also found during intensified hospital-based surveillance for pertussis complications in children under 16 years in 1997–1998 [59]. Possible explanations could be a lower hospitalization threshold in FEG, perhaps due to a lower density of physicians in private practice than in FWG [60] or a higher degree of awareness for the diagnosis.

Sentinel surveillance in two cities in FWG and FEG [40] suggests that pertussis incidence in the two parts of Germany is similar in adults despite the lack of childhood vaccination in FWG prior to 1991. Adults in FEG vaccinated in childhood presumably experienced less natural boostering than non-vaccinated adults in FWG, who would have experienced natural infection – thought to induce longer lasting immunity than vaccination – in childhood more frequently than in FEG. Thus, FEG adults might be expected to be more susceptible to pertussis than FWG adults. This could explain the similar incidence in adults in the two parts of Germany despite evidence for a higher level of *Bordetella pertussis *transmission in FWG. This is corroborated by a seroprevalence study performed in several European countries including FWG and FEG in the mid-1990s [61,62], which revealed that overall, adults were less likely to have high pertussis-toxin antibody (anti-PT) concentrations suggestive of recent acute infection than younger persons. However, in countries with a history of high vaccination coverage (The Netherlands, Finland and FEG), adults comprised a higher proportion of cases with recent pertussis infection than in countries with low vaccination coverage (France, FWG, United Kingdom, and Italy). Thus, in the mid-1990s adults were 0.6 times less likely to have high anti-PT than children and adolescents in FEG, but 0.3 times less likely in FWG. While this difference in age distribution in the two parts of Germany has likely narrowed, the markedly higher incidence of hospitalizations for pertussis in infants and lower vaccination coverage among adolescents in FWG suggest that differences in epidemiologic pattern persist. Furthermore, differences in contact patters, another determinant of infectious disease transmission [63], likely also exist. For instance, one difference between FEG and FWG in this regard is the higher proportion of children attending day care in FEG, even close to 20 years after reunification. In FEG states, day care attendance of children < 3 years of age in 2007 ranged from 31.7% in Saxony to 51.4% in Saxony-Anhalt, but from 5,2% to 36,1% in FWG states [64], with less extreme differences in older children. Such differences could influence disease transmission patterns between children as well as from children to adults. However, this remains hypothetical without robust surveillance data from both parts of Germany.

## Conclusion

Nationwide pertussis reporting is urgently required to better understand disease transmission patterns, for early recognition, control and prevention of outbreaks and for the evaluation of vaccination policy. Universal recommendation for an additional pertussis booster vaccination at the age of 5 to 6 years since 2006 is expected to decrease morbidity in older children. In keeping with recommendations of the Global Pertussis Initiative [65], improved uptake of the recommended adolescent booster as well as vaccination of persons with close contact to infants is crucial to further reduce morbidity. The high proportion of cases in adults suggests a need for additional booster vaccinations.

## Competing interests

The authors declare that they have no competing interests.

## Authors' contributions

WH performed the literature searches, collated the data from the different sources, performed most of the data analysis and drafted the manuscript. DB, EJ, ML, HO provided and contributed to the analysis of routine surveillance data, SR performed analysis of the school entry vaccination coverage data, and all authors contributed to the interpretation of the results and to the final version of the manuscript.

## Pre-publication history

The pre-publication history for this paper can be accessed here:

http://www.biomedcentral.com/1471-2334/9/22/prepub

## Supplementary Material

Additional file 1 **Pertussis vaccination and surveillance practices, vaccination coverage and disease burden in former East and West Germany, 1964–2007.** Summary of pertussis vaccination and surveillance practices, vaccination coverage and disease burden in former East and West Germany, 1964–2007 Click here for file
