# Supplementary material for: The epidemiology of pertussis in Germany: past and present
Source: BMC Infect Dis. 2009 Feb 25;9:22. doi: 10.1186/1471-2334-9-22 (PMC2653494; doi:10.1186/1471-2334-9-22)
Supplement: Additional file 1 — Pertussis vaccination and surveillance practices, vaccination coverage and disease burden in former East and West Germany, 1964–2007. Summary of pertussis vaccination and surveillance practices, vaccination coverage and disease burden in former East and West Germany, 1964–2007 [file 1471-2334-9-22-S1.doc]

Table 1: Pertussis vaccination and surveillance practices, vaccination coverage and disease burden in former East and West Germany, 1964-2007

|  |  | **1964-1990** | **1991-2000** | **2001-2007** |
| --- | --- | --- | --- | --- |
| **Pertussis vaccination**  **recommendations** | FEG | Mandatory: 3 vaccine doses in 1^st^ and booster in 3^rd^ year of life (1964-1990) | 1991: All infants  1993: Catch-up by age 6  2000: Adolescent booster | 2001: Persons with occupational contact to children  2004: Cocoon strategy  2006: Preschool booster |
|  | FWG | 1969-1974: Routine infant vaccination  1975-1991: High risk children < 2 years |  |  |
| **Vaccine type** | FEG | Whole cell vaccine | Whole cell vaccine, acellular vaccine available 1995 | Acellular vaccinesrecommended |
|  | FWG |  |  |  |
| **Childhood vaccination coverage (%)** | FEG | >90% | 1994, 2-year old children: 4 doses 46.3%, ≥ 1 dose: 92.5% | 1998-2006, 4-6 year old children: increase from 85.9% to 95.9% |
|  | FWG | 2-60% | 1994, 2-year old children: 4 doses 46.6%; ≥ 1 dose 76.7% | 1998-2006, 4-6 year old children: increase from 57.7% to 92.2% |
| **Notification** | FEG | Statutory surveillance,  aggregate data available | Statutory surveillance, case-based data available for 1995-2000 | Statutory surveillance, case-based data available |
|  | FWG | Not notifiable | Not notifiable | Not notifiable |
| **Notified pertussis**  **(cases/100,000 inhabitants)** | FEG | <1 (by late 1980s) | 2000: 20.5 | 2007: 39.3 |
| **Estimated incidence (cases/100.000 inhabitants)** | FWG | 160-180 (based on surveys) | ‑ | ‑ |
| **Sentinel surveillance in adults (estimated cases/100.000 inhabitants)** | FEG | ‑ | ‑ | 2002-2004: 160 |
|  | FWG | ‑ | ‑ | 2002-2004: 169 |
| **Hospitalized infants with pertussis /year/100,000 infants** | FEG | No data | No age-specific data | 2002-2007: Range 21.8-61.3 |
|  | FWG |  |  | 2002-2007: Range 68.0-115.2 |
